# Rejuvant®, a potential life-extending compound formulation with alpha-ketoglutarate and vitamins, conferred an average 8 year reduction in biological aging, after an average of 7 months of use, in the TruAge DNA methylation test

**DOI:** 10.18632/aging.203736

**Published:** 2021-11-30

**Authors:** Oleksandr Demidenko, Diogo Barardo, Valery Budovskii, Robb Finnemore, Francis R. Palmer, Brian K. Kennedy, Yelena V. Budovskaya

**Affiliations:** 1 TruMe Inc., Alameda, CA 94502, USA; 2 Healthy Longevity Translational Research Programme, Yong Loo Lin School of Medicine, National University Singapore, Singapore 117456, Singapore; 3 Ponce de Leon Health, Fernandina, FL 32034, USA; 4 Centre for Healthy Longevity, National University Health System, Singapore 117456, Singapore; 5 Singapore Institute for Clinical Sciences, A*STAR, Singapore 117609, Singapore

**Keywords:** aging, DNA methylation, alpha-ketoglutarate (AKG), biologic age, Rejuvant

## Abstract

The search continues for possible interventions that delay and/or reverse biological aging, resulting in extended healthspan and lifespan. Interventions delaying aging in animal models are well established; however, most lack validation in humans. The length of human lifespan makes it impractical to perform survival analysis. Instead, aging biomarkers, such as DNA methylation (DNAm) clocks, have been developed to monitor biological age. Herein we report a retrospective analysis of DNA methylation age in 42 individuals taking Rejuvant®, an alpha-ketoglutarate based formulation, for an average period of 7 months. DNAm testing was performed at baseline and by the end of treatment with Rejuvant® supplementation. Remarkably, individuals showed an average decrease in biological aging of 8 years (p-value=6.538x10^-12^). Furthermore, the supplementation with Rejuvant® is robust to individual differences, as indicated by the fact that a large majority of participants decreased their biological age. Moreover, we found that Rejuvant® is of additional benefit to chronologically and biologically older individuals. While continued testing, particularly in a placebo-controlled design, is required, the nearly 8-year reversal in the biological age of individuals taking Rejuvant® for 4 to 10 months is noteworthy, making the natural product cocktail an intriguing candidate to affect human aging.

## INTRODUCTION

Aging is a near universal biological process that manifests as a general decline in health and vitality, eventually leading to death. Aging is associated with the development of a wide range of chronic diseases, including cancer, Alzheimer's, diabetes, cardiovascular disease and many other conditions [1, 2]. If aging can be delayed, chronic disease onset will be forestalled, functional capacity maintained and, in all likelihood, complications due to infectious diseases, such as Covid-19 and influenza, reduced [3]. In short, humans will have a longer healthspan and lifespan.

Aging is typically measured chronologically in days or years, with median human survival on the order of eight decades. If we hope to control the aging process, we need to learn how to measure the rate of aging in shorter time periods. Moreover, aging progresses at different rates in different individuals. Our true biological age is influenced by many additional factors, such as genetic background, lifestyle, and disease. To address this challenge, several biological markers of aging have been developed. These markers are unique sets of molecules or changes in the epigenetic state of an individual's DNA that reflect their current aging status [4–6]. Among the most promising biomarkers of the aging process are DNA methylation patterns. DNA methylation is an epigenetic mechanism that plays an important role in the regulation of gene expression, organism development and disease [7, 8].

Methylation of lysine residues within core histones, H3 and H4, initiates a conformational modification in the chromatin structure that is associated with changes in transcriptional activity. However, the most widely studied epigenetic mark is the direct methylation of DNA itself. This modification involves the conversion of cytosine to 5’-methylcytosine, catalyzed by DNA methyltransferases, and typically occurs within the CpG dinucleotide sequences (CpGs). These CpG sequences, clustered in regions known as CpG islands (CGIs), are most often found in promoters of housekeeping genes [9, 10]. It has been shown that hypermethylation of CpG islands is linked with transcriptional silencing, whereas demethylated CpG islands are more often found during embryogenesis and serve as a hallmark of actively transcribed genes. During aging, two types of changes in DNA methylation have been observed and carefully characterized: (1) epigenetic drift, or progressive stochastic changes in DNA methylation patterns between individuals that occurs with increasing age [11], and (2) the epigenetic clock – a DNA methylation-derived measure that is highly correlated with chronological age and proposed to measure biological age [9, 12, 13].

The epigenetic clock is an attractive biomarker of aging because it applies to most human tissues, capturing aspects of biological age such as frailty [14], cognitive/physical fitness in the elderly [15], age-acceleration in obesity [16], premature aging in Down’s syndrome [17] and HIV infection [18], Parkinson’s [19] and Alzheimer’s disease-related neuropathologies [20], as well as cancer [21] and lifetime stress [22]. Markers of biological aging represent an important tool to clinically validate the effects of longevity-based interventions. For the first time, these biomarkers of aging give scientists the opportunity to study the effects of anti-aging compounds in real-time and directly in humans. One of the most promising anti-aging compounds discovered to date is Alpha-Ketoglutarate (AKG) [23].

AKG is an endogenous intermediary metabolite in the Krebs cycle whose levels naturally decline during aging. AKG is involved in multiple metabolic and cellular pathways. These include functioning as a (an) signaling molecule, energy donor, precursor in the amino acid biosynthesis, and a regulator of epigenetic processes and cellular signaling via protein binding [24–26]. AKG deficiency in stem and progenitor cells increases with age [27]. As animals age, mitochondrial function is progressively impaired and cellular metabolic flux in the mitochondria declines, which exacerbates AKG deficiency. Chin et al. reported that AKG increased the lifespan of *C. elegans* [28]. Building on these results, AKG (and calcium salt) combined with other Generally Recognized as Safe (GRAS) compounds were studied in mice. The non-genetically altered mouse is the preferred mammalian model to study aging, since the biochemical processes involved in mice aging may apply to other mammals, including humans [29]. In a recent study, sponsored by Ponce de Leon Health and performed at the Buck Institute for Research on Aging, the effect of alpha-ketoglutarate (delivered in the form of a calcium salt - CaAKG) on healthspan and lifespan in C57BL/6 mice was reported. The authors showed that in the mice, AKG reduced frailty and enhanced longevity, indicating a compression of morbidity [23]. These and other discoveries suggest that AKG may be an ideal candidate for pro-longevity human studies.

In this study, we examined the cross-sectional and longitudinal association between the epigenetic clock, health status, physical fitness and the effects of taking Rejuvant® (sustained release CaAKG + a specific vitamin depending on sex) on human biological aging. We followed 42 self-reported healthy individuals who had taken AKG supplementation for a period of 4 to 10 months. The effects of AKG on biological aging, and the possible correlation of other physiological effects, are discussed.

## RESULTS

For this study, we reviewed 42 participants who had elected to take Rejuvant® for a period of 4 to 10 months. All participants were actively on the Rejuvant® product. The need to follow this study with a placebo-controlled study is described in the Discussion. The general characteristics of this cohort are described in Table 1. All participants reported good or excellent health status without any chronic medical conditions. Only one participant was a smoker at baseline and continued to smoke throughout the study. The majority of the study participants (66.7%, n = 28) were male. The mean chronological age of this cohort was approximately 63 years old.

**Table 1:** Descriptive characteristics of the study participants.

Total Participants	42
Gender (Female/ Male)	14/ 28
Female:	
Chronological Age (median; range)	64.09; 43.49 to 72.46
Biological Age at Baseline (median; range)	62.15; 46.4 to 73
Biological Age at T7* (median; range)	55.55; 33.4 to 63.7
Male:	
Chronological Age (median; range)	62.78; 41.31 to 79.57
Biological Age at Baseline (median; range)	61.85; 41.9 to 79.7
Biological Age at T7* (median; range)	53.3; 33 to 74.9

*Indicates biological age as measured by TruMe test after an average of seven months of treatment.

For all 42 participants, we were able to measure the baseline biological age using the TruMe age prediction model before they began taking Rejuvant®. We utilized the TruAge prediction model with Sanger sequencing for DNA methylation analysis. In total, 3 genes including 9 CpG sites were analyzed by the Sanger sequencing. The DNA methylation values obtained for all CpG sites were included in the TruMe age-prediction model (pending publication).

**Figure 1:**
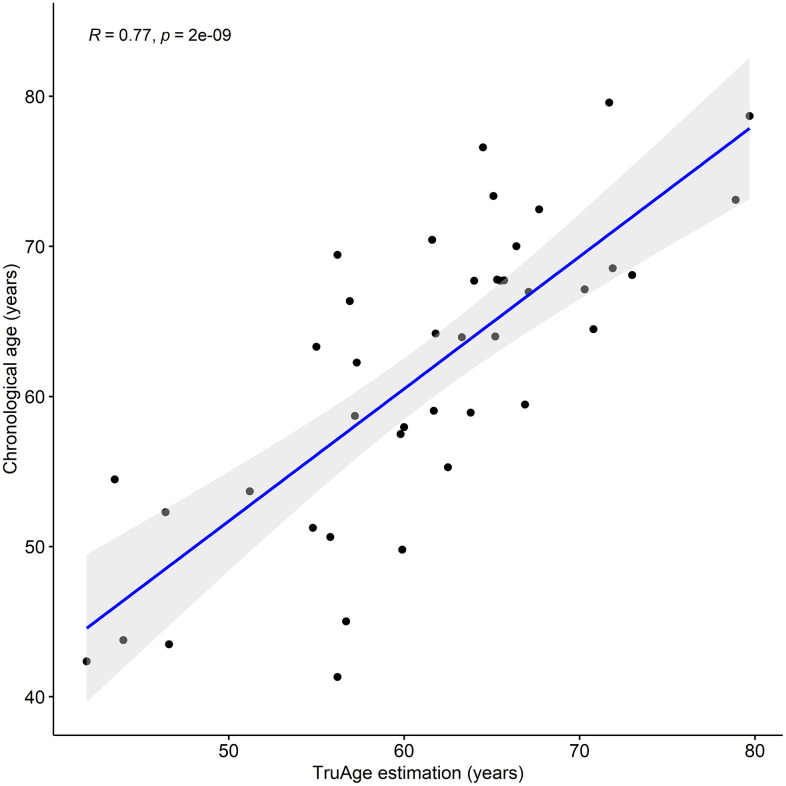
**TruAge age estimation and baseline chronological age are linearly correlated.** The TruAge biological age is highly (adjusted R-squared = 0.77) statistically significantly (p-value = 2x10^-9^) correlated with chronological age of the 42 patients.

We assessed the prediction error of the TruAge epigenetic test, as previously described. Comparison of the predicted vs. actual age values yields a median absolute error of 4.23 years. The mean error of 0.35 years shows that this population as a whole may be slightly younger than expected. It is known that pre-processing normalization of DNA methylation datasets and their age variance can bias the difference between the estimated and chronological age. A measure of robustness to these factors involves calculating the residuals of a linear regression of predicted vs. actual age. We found the estimated age to have a regression coefficient of 0.88 and that the linear regression (line fitted in Figure 1) displayed an adjusted R-squared of 0.59 with a median absolute error of 3.97 years. Finally, we also found a statistically significant (p-value=2.026x10^-9^) Pearson linear correlation of 0.77 (95% CI: 0.61 to 0.87) between our cohort's estimated and actual age at baseline.

**Figure 2:**
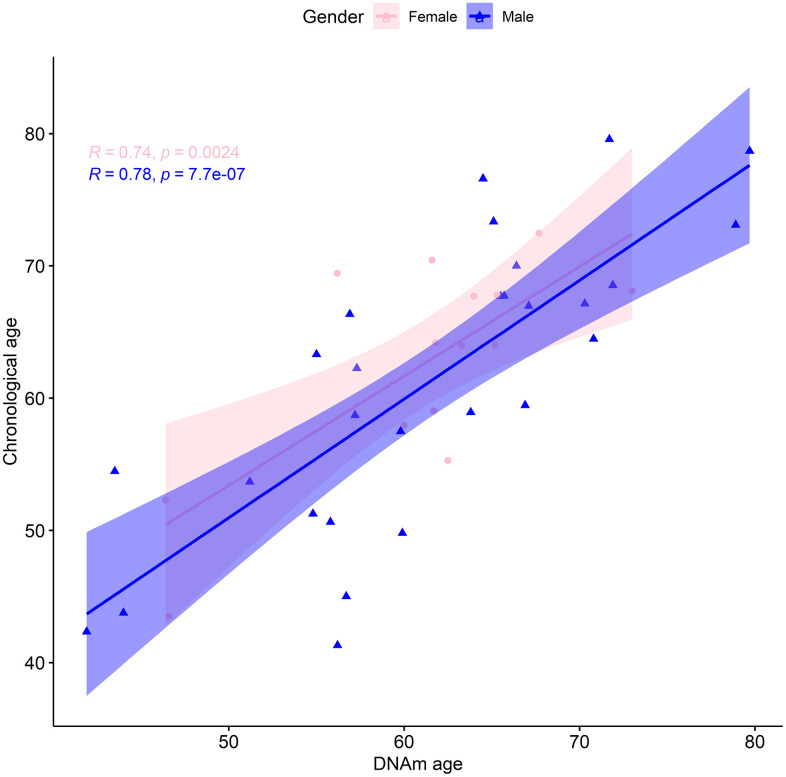
**TruAge age estimation and baseline chronological age are linearly correlated, in both genders.** The x-axis depicts DNA methylation age estimated by TruAge, and the y-axis the chronological age of the 42 patients at baseline. The pink and blue linear correlation plots depict the similarly high statistically significant linear correlation between both axes, for female and male patients, respectively.

We also considered whether there was a sex bias of the biological age assessment by analyzing males (28) and females (14) separately. Following the same procedure as before, for males we found a median absolute error of the predicted vs actual age of 4.92 years, and a mean error of -0.22 years. The median absolute error of the linear regression was 4.64 years, with an adjusted R-squared of 0.6 and a regression coefficient of 0.90 (Figure 2). Additionally, estimated and actual age at baseline for males were linearly correlated (p-value=7.74x10^-7^) with a magnitude of 0.78 (95% CI: 0.58 to 0.9). For females the analogous regression procedure displayed a median absolute error of 3.887 years, with an adjusted R-square of 0.51 and a regression coefficient of 0.83 (Figure 2). Furthermore, we found a median absolute error of the predicted vs actual age of 3.41 years. The mean error of 1.48 years could indicate that the females of our cohort are likely to be younger than expected. To explore this, we applied a multilinear regression with gender as an additional variable to our entire cohort. Gender was not found to be a statistically significant predictor of chronological age at baseline (Table 2). Moreover, there was a statistically significant (p-value=0.002) linear correlation of 0.74 (95% CI: 0.35 to 0.91) between the estimated and real age at baseline for females. In conclusion, within the context of this limited dataset, there was no statistical difference in the predictive ability of the TruAge epigenetic test with respect to males and females.

**Table 2:** Effect of Rejuvant® on biological age of study participants.

**Biological age distributions**	**Male participants (in years)**	**Female participants (in years)**
Minimum Biological Age at Baseline	41.9	46.4
Median Biological Age at Baseline	61.85	62.15
Mean Biological Age at Baseline	61.38	61.09
Maximum Biological Age at Baseline	79.7	73
Minimum Biological Age at T7*	33	33.4
Median Biological Age at T7*	53.3	55.55
Mean Biological Age at T7*	53.53	54.66
Maximum Biological Age at T7*	74.9	63.7
Minimum change in Biological Age	-1.86	-0.41
Median change in Biological Age	7.09	5.83
Mean change in Biological Age	8.44	6.98
Maximum change in Biological Age	22.7	14.09

*Indicates biological age as measured by TruMe test after an average of seven months of treatment.

Participants completed a survey at the start and end of the trial. This self-reported questionnaire included information about diet, alcohol intake, previous consumption of Rejuvant®, health, height and weight (which allowed for estimation of BMI), sleep duration, smoking status, exercise frequency, physical activity level, meal frequency, snacking frequency, number of additional dietary supplements consumed and frequency, hair status, education, healthy lifestyle mindset and trust in dietary supplements (Supplementary Table 1).

We leveraged baseline survey information to check if there were other confounders in our cohort, performing a multivariable linear regression. Due to the considerable number of predicative variables, we performed a stepwise linear regression. Although there were additional covariates selected in the stepwise model, when this was compared, by means of analysis of variance, with the simpler univariate linear regression using solely the TruAge estimated age, the obtained p-value was 0.12 (Supplementary Table 2). This indicates that in terms of comparison, the two models are not statistically significantly different, and, therefore, the simpler model was selected.

It was of interest to determine whether the difference between chronological age and TruAge is related to lifestyle or demographic factors. In other words, it is important to assess if these covariates are associated with biologically younger or older individuals. We selected BMI, alcohol consumption, self-assessed health, sleep duration, smoking history, exercise frequency and intensity, and hair abundance as predictor variables, since this subset of the survey information collected may clearly be associated with and/or influence biological age. The stepwise linear regression did not find any statistically significant association between any of these variables with the difference between chronological and TruAge biological age (Supplementary Table 2). Of note, the number of participants is quite small (for instance, there was one smoker, and only 6 who reported a history of smoking). Therefore, it is likely that one or more of these lifestyle parameters influence the biological age as measured by the TruAge test and that a larger dataset would uncover associations.

### Results of the CaAKG consumption on biological age

The goal of the study was to determine the effect of Rejuvant® supplementation on human biological aging by measuring DNA methylation. Following the baseline measurement, each study participant was supplied Rejuvant® for the duration of the study. Even though the participants were advised to use the treatment for 4 to 6 months, there was considerably individual differences in treatment duration. Upon completion of the self-chosen treatment period, participants submitted their saliva samples for analysis of their biological age using commercially available TruMe tests.

Based on the questionnaires submitted at baseline and the end of the trial, we identified a subset of 13 individuals who reported no changes in diet type, drinking frequency, additional dietary supplements intake, sleep duration and exercise frequency. This homogeneous subset was therefore used for the preliminary assessment of the independent effect of CaAKG, as the other covariates are controlled for by design.

**Figure 3:**
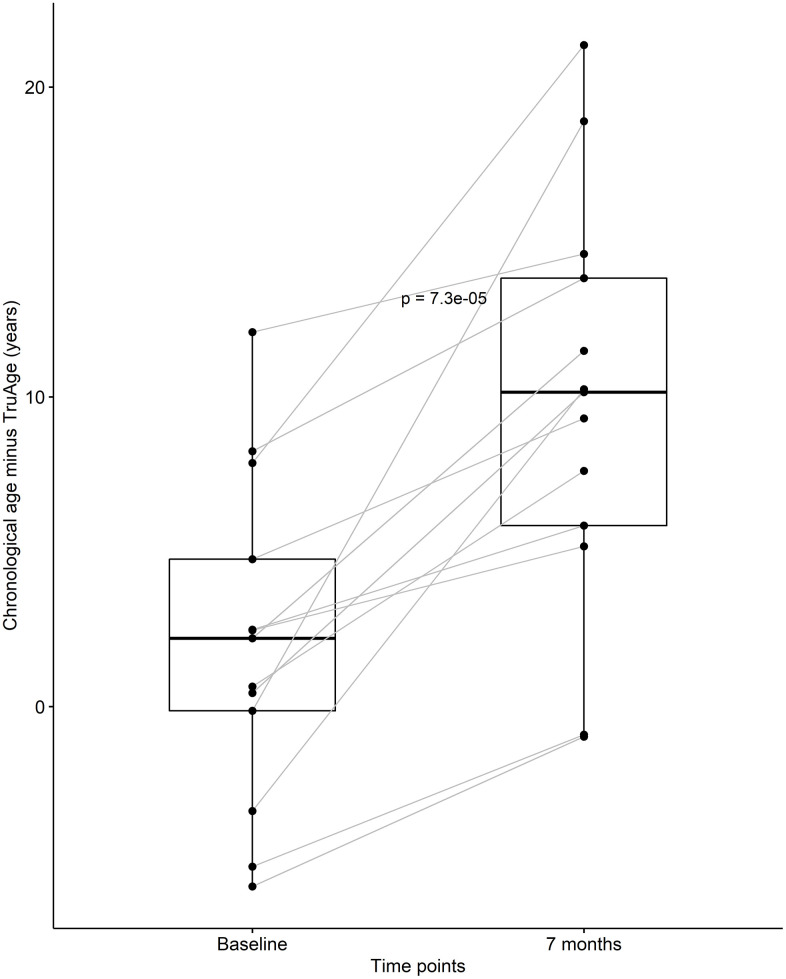
**CaAKG decreased methylation age among a homogeneous sub-population.** The paired box plots represent the treatment effects at the patient and group level (n=13), between baseline and end of the trial (which on average had a duration of 7 months). The box plots depict the median and the 25^th^ and 75^th^ quartiles.

At baseline, this subset or our cohort was on average 2.06 years biologically younger than their chronological age. By the end of the treatment this sub-population was on average 9.74 years biologically younger than their respective chronological age. Using one-sided Welch two sample paired t-test, this difference in means of 7.69 years was found to be statistically significant, with a p-value of 7.263x10^-5^ (Figure 3). It is noteworthy that every subject in this small group decreased their biological age.

**Figure 4:**
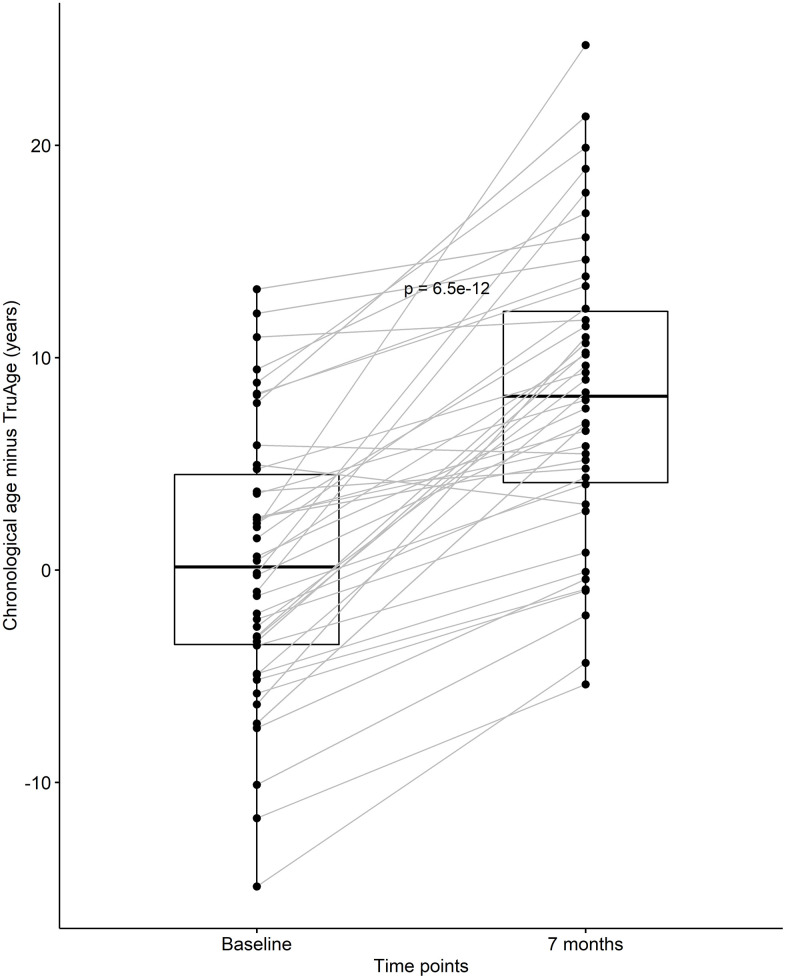
**CaAKG decreased methylation age in the entire cohort.** The paired box plots represent the treatment effects at the patient and group level (n=42), between baseline and end of the trial (which on average had a duration of 7 months). The box plots depict the median and the 25^th^ and 75^th^ quartiles.

After discovering that CaAKG supplementation consistently decreased epigenetic age in a small homogenous population, we continued by assessing its effects on the entire cohort of 42 patients. At baseline, the cohort was on average 0.35 years biologically younger than their chronological age. By the end of the treatment, this value changed to an average of 8.31 years biologically younger than their respective chronological age. Using one-sided Welch two sample paired t-test, this difference in means of 7.96 years was found to be statistically significant, with a p-value of 6.538x10^-12^ (Figure 4).

**Figure 5:**
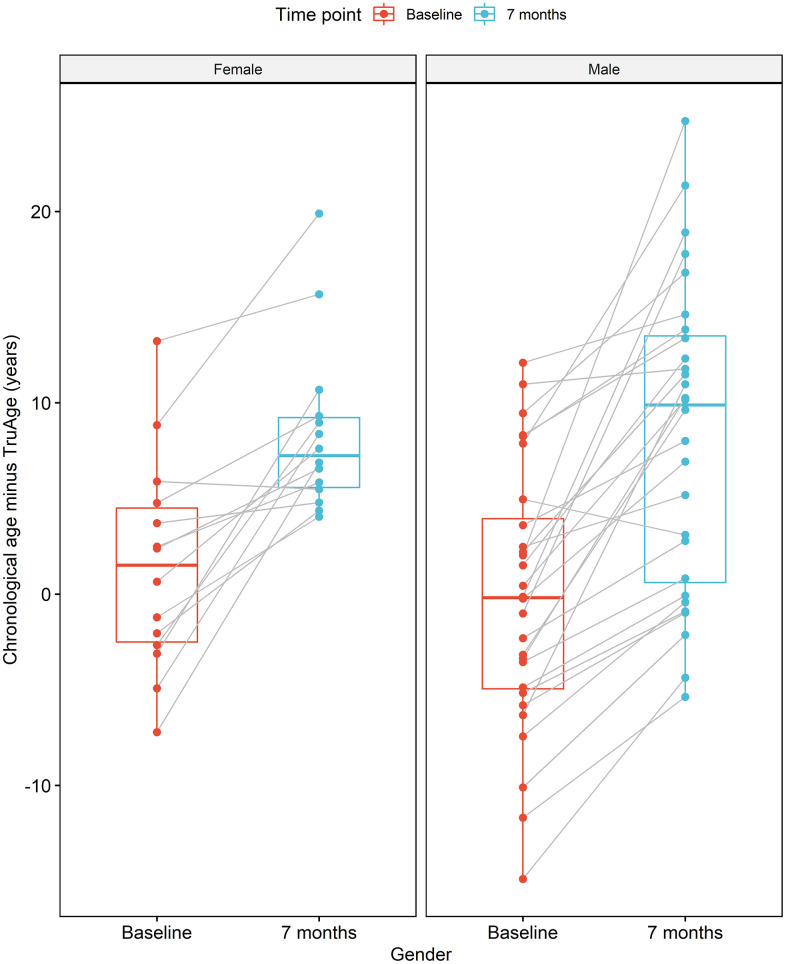
**CaAKG decreases methylation age regardless of gender.** The image displays the effects of CaAKG on methylation age between the start and end of treatment, broken down by gender. For each gender, the red and blue paired box plots, depict the data at baseline and endpoint, respectively. The boxplots are based on the median and the 25^th^ and 75^th^ quartiles.

The formulation of the Rejuvant® supplement is itself different for males and females. More specifically, in addition to CaAKG, vitamin A and vitamin D are added to the men’s or women’s commercial formulation, respectively (see Methods). Regardless, the effect of supplementation with Rejuvant® decreases epigenetic age in a statistically significant manner in males and females (Figure 5). The paired mean decrease is 8.44 years in males and 6.98 years in females. Only 2 individuals (1 male and 1 female) exhibited a slightly increase in biological age.

The validity of the statistical analysis using the entire cohort is predicated on covariate modelling, for the detection of variables that result in a statistically significant difference in the TruMe DNA methylation test, we used stepwise regressions iteratively. This procedure culminated in the most parsimonious model using only baseline chronological age, and the difference between baseline chronological and biological ages, as the only statistically significant predictors of the magnitude of the decrease in epigenetic age by Rejuvant®. Accordingly, we modelled the effect of Rejuvant® supplementation on methylation age as a linear regression of these two variables. These data indicate that those people with higher biological age (relative to their chronological age) and/or people with high baseline chronological age have the largest response to Rejuvant®.

**Figure 6:**
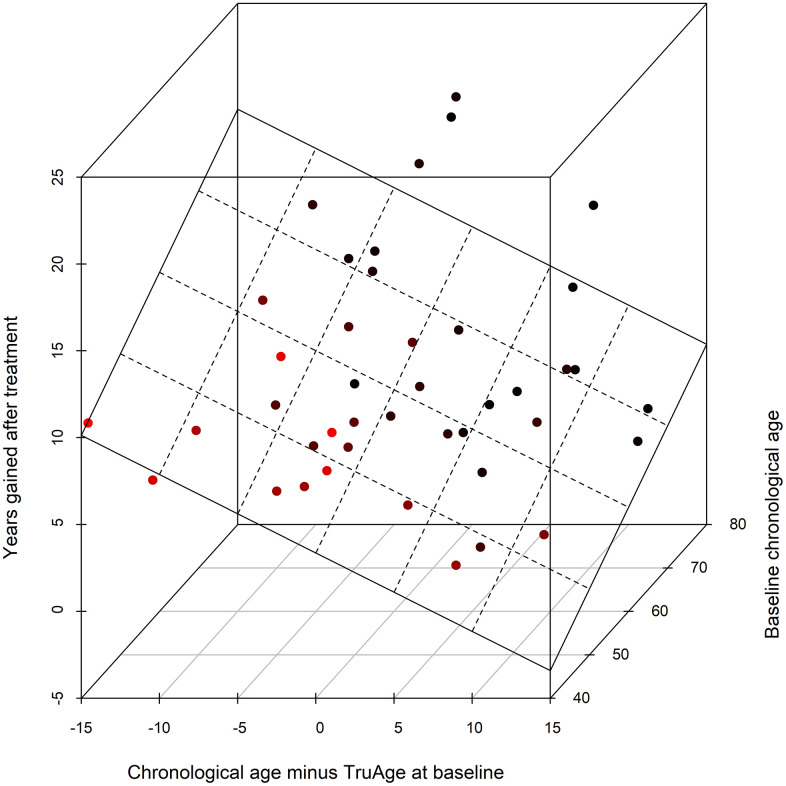
**3D scatter plot of the covariates influencing CaAKG treatment efficacy.** The x-axis refers to the difference, in years, between the chronological and biological ages at baseline (a patient in an older state will have larger positive value). The y-axis depicts the effect of CaAKG treatment in terms of years that the methylation age decreased (higher values indicate larger benefits). The z-axis is the chronological age at baseline (older patients will be “deeper”). The 3D plane was traced by using the linear regression of both covariates to treatment effect.

Using the final reduced model, with only the two variables found to be statistically significantly related with a decrease in epigenetic age over the course of the treatment, the epigenetic age difference (in years) of about 7 months of CaAKG supplementation can be modeled by the equation:


Decrease in TruAge= −5.41+0.22×age−0.45×diffAge

with “age” standing for chronological age at the start of the treatment and “diffAge” defined as the difference between chronological age and TruMe age at baseline. The plane representing this regression is depicted (Figure 6).

Interestingly, duration of the treatment is not correlated with the magnitude of the effect of Rejuvant® supplementation. This indicates that the median treatment duration of our cohort, approximately 7 months, may be sufficient to saturate the benefits of the supplementation.

Interestingly, this finding is consistent with mathematical extrapolations from a large-scale study of complete blood count measurements, which forecast that the effects of a longevity intervention in a healthy population will saturate in a short period of time [30]. This same study predicted that the benefits of such interventions would be greater in individuals closer to a frail state, consistent with our data. Having said this, there remains very little data on longer term use of Rejuvant® or higher doses of the product, and further effects on biological age are feasible. We also did not measure whether the benefits in biological age are lost if the supplement is discontinued, therefore, continual supplementation may be needed to maintain any effects observed.

## DISCUSSION

AKG has been shown to extend lifespan in various model systems. In this study, we used a previously developed algorithm that predicts human biological age to determine if Rejuvant®, sustained release CaAKG + vitamins has a beneficial effect on human longevity. A total of 42 individuals, known to be taking Rejuvant® and who had submitted saliva samples for DNA methylation testing, were selected to participate in a customer biological aging result survey and analysis. Their DNAm TruAge Index had been measured at baseline (before starting Rejuvant®) and retested after an average of 7 months of use. Overall, these 42 individuals showed statistically significant average reduction in their biological age of approximately 8 years.

General thinking in the aging research field is that interventions are likely to affect subsets of the population, and no one intervention (lifestyle or small molecule) will delay or reduce biological age in the entire population. Surprisingly, in this group the vast majority of participants responded with a reduced biological age after Rejuvant® treatment. While the study does have limitations (described below), these findings are encouraging. Interestingly, there were two parameters that influenced the magnitude of the response: those participants with higher biologic age relative to chronological age and those with higher chronological age at baseline. This suggests, perhaps contrary to expectations given the known role of AKG in augmenting exercise performance, that Rejuvant® has a larger response in participants biologically older than their chronological age. One might also predict this outcome for a longevity intervention on the basis of the hypothesis that individuals with low relative biological age are already undergoing near optimized aging and have less to gain. Currently, there is insufficient data on human aging to predict which populations will respond to a particular intervention.

The TruAge methylation test, which remains proprietary, was developed by examining a limited number of methylation sites in CpG islands of promoters, based on optimization to chronological age using a machine learning approach. While it surveys a smaller portion of the genome than other methylation clocks, it has the advantage of being more affordable. In addition, TruAge test is easily used by consumers, who place saliva on a paper card and mail in the sample for analysis. The TruAge test was shown to report similar results when compared to other epigenetic clocks (unpublished), yet further testing using other methylation clocks and different biomarkers of aging would be beneficial to measure the effects of Rejuvant® on human longevity. A fundamental question regarding different biological aging measures relates to their level of concordance: do they measure the same, overlapping or completely different aspects of the aging process?

The data in this study, while limited, suggests that CaAKG may indeed impact aging, at least as measured by methylation. It is also worth noting that AKG is a known substrate for DNA demethylases [31], which potentially demethylate DNA sites interrogated by TruAge. However, the AKG supplementation leads to both demethylation and hyper methylation of some CpG sites in saliva cells, suggesting that Rejuvant® may have a larger effect on methylation-based aging clocks than other indicators of biologic age.

There are several limitations to this study. Primarily, it is not placebo controlled. Therefore, one potential concern is that the placebo effect may have contributed to some extent to the changes observed. However, the self-reported trust in the efficacy of dietary supplements was not deemed a statistically significant predictor in any of our regression models, which mitigates risk to an extent. Moreover, the study describes a limited sample size and we were unable to collect other kinds of data relevant to aging, for instance clinical markers of aging and disease, and apply other biological aging clocks [6]. Future randomized clinical trials will be required to confirm the findings presented here. Nevertheless, the results in this manuscript suggest that Rejuvant® may have significant effects on biological age as measured by DNA methylation of saliva samples.

## MATERIALS AND METHODS

### Participants

A group of 42 self-reported healthy individuals (14 females and 28 males) who had submitted saliva samples (two samples per subject). Saliva samples were collected at Baseline (T0) and 4-10 months after the participant began taking Rejuvant® (T7) at a fixed dose of two tablets per day. Each dose contained 1 gram of Calcium Alpha-Ketoglutarate, along with Vitamin A for the male participant’s formulation or Vitamin D for the female participant’s formulation, and delivered in a timed-release formulation, as illustrated in the formulation labels.

TruMe identified prospective study participants from all customers with two previously completed DNAm tests (one baseline before starting and a second test 4 to 10 months Rejuvant(R) supplementation. Prospective subjects were consented and asked to fill out a questionnaire reporting lifestyle changes before and after supplementation in diet, exercise, sleep, alcohol consumption, smoking and nutritional supplement use. The consent form included the following statement: "TruMe would like to have your permission to use your biological age results for scientific and academic purposes. You will always be anonymous and no personally identifiable information will ever be shared. TruMe does not use full genome sequencing and therefore does not generate nor maintain any genetically identifiable data. We never sell your data or personal information." Participants who provided appropriate consent following GCP principles, were included in the study. Analysis of participant data was performed in aggregate and anonymously (Table 1).

**Table:** 

Vitamin A (as retinyl palmitate)	900 mg	100%
Calcium	190 mg	15%
Calcium Alpha-Ketoglutarate Monohydrate (LifeAKG™)	1000 mg	†

**Table:** 

Vitamin D	25 mg
Calcium	190 mg
Calcium Alpha-Ketoglutarate Monohydrate (LifeAKG™)	1000 mg

### Sample collection and bisulfate sequencing

Saliva samples were self-collected by participants at home using commercially available TruMe sampling kits. Participants were instructed to collect about 200-300 mL of their saliva samples onto FTA Classic Cards (FTA Classic Cards, #WB120205, from GE Healthcare Life Sciences). Saliva samples were allowed to air dry for 30-45 minutes, before they were shipped to TruMe Labs.

### DNA methylation and DNAm age calculation

From each sample, 1 inch diameter circles were obtained, and DNA eluted with Quick-DNA Microprep Plus Kit (ZymoResearch, CA, USA) according to the manufacturer’s protocol. 200-500ng of eluted DNA was bisulfite converted with the EZ DNA Methylation-Lighting™ Kit according to the manufacturer’s instructions (ZymoResearch, CA, USA). PCR amplification of bisulfite converted DNA was performed using standard target specific primers (IDT, Newark, NJ, USA). The PCR reaction was set up using ZymoTaq PreMix E2004 (ZymoResearch, CA, USA).

Each PCR fragment was analyzed using the standard Sanger sequencing protocol and methylation levels were analyzed using a proprietary algorithm, developed by TruMe Inc. The TruMe age-prediction algorithm uses a multivariate model to predict biological age of the individual.

### Statistical analysis

The data was analyzed, and the plots generated using the R programming language. Continuous variables were tested for normality using the Shapiro-Wilk test. In cases, where we fail to reject the null hypothesis (p-value not less than 0.05) the one-sided Welch two sample paired t-test was used, otherwise the one-sided Wilcoxon signed rank paired test with continuity correction was deployed [32]. Figures 1–5 were done using the ‘ggpubr’ R package. Figure 6 was created using the ‘scatterplot3d’ R package [33].

## Supplementary Material

Supplementary Table 1

Supplementary Table 2
