# Supplementary material for: Rejuvant®, a potential life-extending compound formulation with alpha-ketoglutarate and vitamins, conferred an average 8 year reduction in biological aging, after an average of 7 months of use, in the TruAge DNA methylation test
Source: Aging (Albany NY). 2021 Nov 30;13(22):24485–99. doi: 10.18632/aging.203736 (PMC8660611; doi:10.18632/aging.203736)
Supplement: Supplementary Table 1 [file aging-13-203736-s001.pdf]

SUPPLEMENTARY TABLE

Supplementary Table 1. Characteristics of participants at baseline.

	Female	Male
n	14	28
Ethnicity (%)		
Asian	1 (7.1)	1 (3.6)
Hispanic/Latino	1 (7.1)	1 (3.6)
White/Caucasian	12 (85.7)	23 (82.1)
White/Caucasian, American Indian or Alaskan Native	0 (0.0)	2 (7.1)
White/Caucasian, Native Hawaiian or other Pacific islander	0 (0.0)	1 (3.6)
Chronological Age T0 (mean (SD))	62.57 (8.11)	61.16 (11.03)
Chronological Age T7* (mean (SD))	63.12 (8.11)	61.76 (11.01)
Biological Age T0 (mean (SD))	61.09 (7.27)	61.38 (9.64)
Biological Age T7* (mean (SD))	54.66 (8.17)	53.53 (8.62)
Weekly alcohol consumption in drinks (%)		
0	5 (35.7)	5 (17.9)
1 to 4	2 (14.3)	10 (35.7)
5 to 8	6 (42.9)	8 (28.6)
9 to 14	1 (7.1)	5 (17.9)
Current number of dietary supplements (%)		
0	2 (14.3)	4 (14.3)
1 to 2	4 (28.6)	3 (10.7)
3 to 4	5 (35.7)	6 (21.4)
5	3 (21.4)	15 (53.6)
Self-rated general health (%)		
Moderate	1 (7.1)	1 (3.6)
Good	7 (50.0)	9 (32.1)
Very good	6 (42.9)	18 (64.3)
Sleep duration (%)		
4-6 hours	1 (7.1)	2 (7.1)
7-8 hours	13 (92.9)	24 (85.7)
More than 8 hours	0 (0.0)	2 (7.1)
Smoking history (%)		
Past smoker but quit	3 (21.4)	3 (10.7)
Never smoker		
Weekly exercise frequency (%)		
Rarely	1 (7.1)	0 (0.0)
1-2 times/week	0 (0.0)	5 (17.9)
3+ times/week	7 (50.0)	17 (60.7)
Daily	6 (42.9)	6 (21.4)
BMI (mean (SD))	21.84 (2.60)	25.70 (3.97)
Days of treatment (mean (SD))	200.57 (35.78)	218.36 (41.57)
Education (%)		
High school diploma (or GED)	1 (7.1)	0 (0.0)
Some college	3 (21.4)	4 (14.3)
4-year college degree	2 (14.3)	10 (35.7)
Graduate-level degree	8 (57.1)	14 (50.0)
Average number of daily meals (%)		
2	6 (42.9)	11 (39.3)
3	7 (50.0)	14 (50.0)
4 or more	1 (7.1)	3 (10.7)
Number of daily snacks (%)		
1	6 (42.9)	18 (64.3)

2	6 (42.9)	8 (28.6)
3	2 (14.3)	2 (7.1)
Opinion about dietary supplements (%)		
Skeptical	2 (14.3)	3 (10.7)
No opinion	2 (14.3)	0 (0.0)
Interested	6 (42.9)	4 (14.3)
Firm believer	4 (28.6)	21 (75.0)
Weekly frequency of dietary supplements (%)		
Never	1 (7.1)	0 (0.0)
Rarely	0 (0.0)	1 (3.6)
Daily	13 (92.9)	27 (96.4)
Healthy living mindset (%)		
Ready to get started	1 (7.1)	1 (3.6)
Practicing	10 (71.4)	15 (53.6)
Dedicated	3 (21.4)	12 (42.9)
Activity level (%)		
Sedentary	1 (7.1)	2 (7.1)
Mildly Active	2 (14.3)	5 (17.9)
Active	5 (35.7)	12 (42.9)
Very Active	6 (42.9)	9 (32.1)
Athletic	0 (0.0)	0 (0.0)
Hair (%)		
Bald	0 (0.0)	4 (14.3)
Thinning	5 (35.7)	11 (39.3)
Thick and/or Full	9 (64.3)	13 (46.4)

*Indicates biologic age as measured by TruMe test after an average of seven months of treatment.
